# Implantable loop recorder migration: Case-based review and implications for clinical practice

**DOI:** 10.1016/j.ahjo.2025.100505

**Published:** 2025-01-30

**Authors:** Allam Harfoush

**Affiliations:** a The Faculty of Health, Medicine, and Society, University of Chester, Chester CH1 4BJ, UK; b The Countess of Chester Hospital, Liverpool Road, Chester CH2 1UL, UK

**Keywords:** Implantable loop recorder migration, Arrhythmia monitoring, Device displacement, Post-implantation complications

## Abstract

**Introduction:**

Implantable loop recorders (ILRs) are vital for continuous rhythm monitoring; however, post-implantation migration can impair device function. ILR migration may range from minor positional shifts causing discomfort to severe displacement, potentially resulting in device malfunction or requiring surgical intervention. This review examines migration patterns to identify factors associated with ILR migration.

**Methods:**

A systematic literature search was conducted in PubMed, Cochrane Library, CINAHL, and EMBASE for case reports on ILR migration from inception to October 2024. Data on patient demographics, comorbidities, device models, implantation sites, detection times, and interventions were qualitatively synthesised to identify factors linked to migration.

**Results:**

Older age, female gender, and specific comorbidities emerged as migration risk factors. Device implantation angulation and depth were common contributors. Migration typically followed a posterior or inferior direction and was detected within 5–35 days, often presenting as loss of connection or continuous chest pain. Migration was also observed following patient manipulation of the device. Although migration is rare, cases requiring video-assisted thoracoscopic surgery (VATS) highlight the significant morbidity associated with this complication.

**Conclusion:**

Optimising implantation techniques and employing effective follow-up strategies can reduce the risk of migration and improve migration detection. Further studies with standardised reporting are needed to better understand this complication.

## 1 Introduction

Implantable loop recorders (ILRs) have become valuable tools in monitoring arrhythmias. Their capacity to provide continuous ECG monitoring over extended periods has transformed patient care by allowing early diagnosis and guiding treatment decisions [1]. However, as with any implanted device, ILRs are not without complications. One clinically significant issue is migration, defined as displacement of the ILR from its intended implantation site. Migration can range from minor shifts causing discomfort to severe displacement, leading to loss of connection, device malfunction, or the need for surgical intervention.

ILRs are primarily implanted in patients with unexplained syncope, cryptogenic stroke, or suspected arrhythmias. Analysis of large cohorts reveals that ILR recipients have a mean age ranging from 56 to 70 years, with most studies reporting a slight male predominance (50–61 %) [[2], [3], [4], [5], [6]]. Preexisting comorbidities, such as hypertension, atrial fibrillation, diabetes, and chronic kidney disease, are common in these patients, affecting 38–53 % in reviewed studies [2,5]. BMI data are inconsistently reported; however, one study indicated an average BMI of 26.39 kg/m^2^, with 20.8 % of patients classified as obese (BMI > 30 kg/m^2^) [6].

While comprehensive data on ILR migration incidence are limited, a study involving 148 participants reported a complication rate of 1.3 %, including a 0.67 % migration rate [7]. This shows the rarity yet clinical significance of this complication, highlighting the need for further exploration of its predictors and implications. This review aims to synthesise case report data on ILR migration, explore predictors, and propose actionable insights to minimise migration risk in clinical practice.

## 2 Methodology

### 2.1 Search strategy

A literature search was conducted to identify case reports and case series detailing incidence of ILR migration following implantation. The search was carried out across multiple electronic databases, including PubMed, Cochrane library, CINAHL, and EMBASE, to ensure a wide capture of relevant cases. Keywords used in the search strategy was (“cardiac loop recorder” OR “implantable loop recorder” OR “ILR” OR “subcutaneous loop recorder”) AND (“migration” OR “displacement” OR “malposition” OR “repositioning” OR “device-related complications” [MeSH]). Filters were applied to include only cases published in English up to October 2024.

### 2.2 Inclusion criteria

Migration was defined as displacement of the ILR identified through imaging or clinical presentation that caused functional impairment (e.g., loss of connection) or significant patient discomfort. Cases were eligible for inclusion if they were published as case reports or case series, and involved patients who experienced ILR migration post-implantation.

### 2.3 Data extraction

Data were systematically extracted from each eligible report using a standardised extraction form on Excel. Key variables included patient demographics (age and gender), device model, device size, time to migration onset, specific characteristics of migration (direction and clinical presentation), and any subsequent management or intervention. Additional factors, such as comorbidities, and follow-up details, were recorded to facilitate the analysis.

### 2.4 Data analysis

Extracted data were analysed descriptively to highlight patterns and trends in ILR migration across cases. Common predictors of migration, including procedural factors, patient characteristics, and device specifications, were synthesised. The outcomes were then assessed to understand the clinical significance of migration and any associated complications or interventions reported.

## 3 Results and discussion

**Fig. 1:**
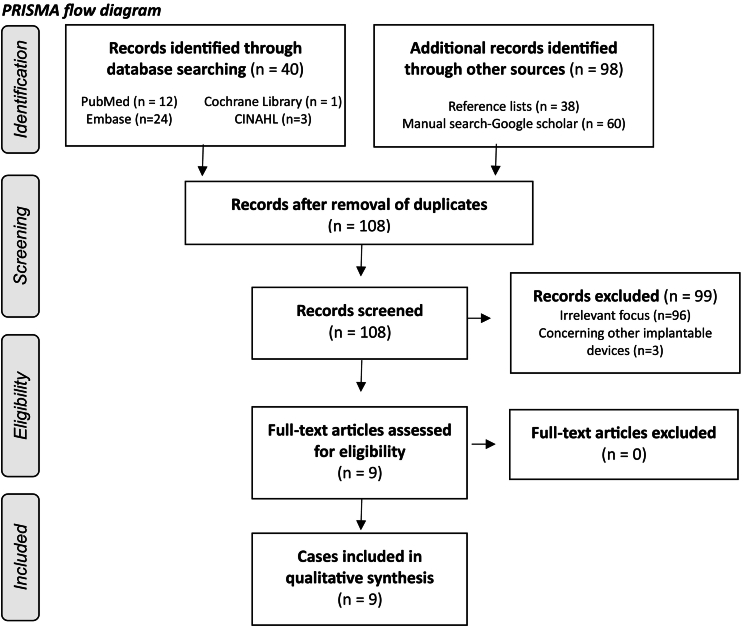
The PRISMA flowchart of this review.

Nine cases were included in this review (Fig. 1). Most cases involved older patients, with an average age above 60. This aligns with broader ILR implantation data, where mean ages range from 56 to 70 years [[2], [3], [4], [5], [6]]. Migration signs presented as loss of signal or connection, sometimes accompanied by chest pain, with detection times ranging from 5 to 35 days post-implantation. Imaging findings highlighted a migration pattern toward the posterior chest. The most common intervention to retrieve the device was video-assisted thoracoscopic surgery (VATS). Table 1 represents the full characteristics.

**Table 1:** The characteristics of the included cases.

References	Age/gender	Comorbidities	BMI	ILR model	ILR dimensions	Implantation site	Implantation depth	Migration signs	Patient manipulation	Diagnosis imaging	Removal	Detection time	Migration pattern	Long term complications
Russo et al. [8]	75 Male	HTN, dyslipidaemia, CKD, COPD, AF	NR	Biomonitor IIIM, Biotronik	77.5 × 8.6 × 4.6 mm	Left anterior chest wall	NR	Lost connection	NR	CXR, CT	VATS	30 days	Anterior costophrenic recess	No
Signore et al. [9]	80 Female	HTN, DM	NR	BioMonitor 3, Biotronik	77.5 × 8.3 × 4.3 mm	Left anterior chest wall	NR	Lost connection	NR	CXR, CT	VATS	7 days	Posteroinferior pleural space	No
Squillace et al. [10]	60 Female	None	19 kg/m^2^	Biomonitor IIIM, Biotronik	77.5 × 8.6 × 4.6 mm	Left 4th intercostal space	Deep toward the intercostal muscle	CP, lost connection	Yes	CXR, CT	VATS	7 days	Left posteroinferior pleural cavity	No
Ang et al. [11]	58 Female	NR	NR	Reveal LINQ LNQ11, Medtronic	44.8 mm × 7.2 mm × 4.0 mm	Left anterior 4th intercostal space	NR	Loss of location	NR	CXR, CT	VATS	28 days	Anterior chest wall	No
Rahkovich et al. [12]	65 Female	HTN, Stroke	NR	BioMonitor 3, Biotronik	77.5 × 8.3 × 4.3 mm	Left 4th intercostal space	Deep toward the intercostal muscle	Lost connection	Yes	CXR, CT	VATS	7 days	Left posteroinferior pleural cavity	No
Basyal et al. [13]	71 Female	B cell lymphoma, pancreatitis	NR	Biotronik ILR		Left 3rd intercostal space	NR	Loss of connection	NR	CXR, CT	Surgery	NR	Left chest wall posterior	No
Hasnie et al. [14]	36 Male	None	NR	Medtronic LINQ	44.8 × 7.2 × 4.0 mm	Left 4th intercostal space	Penetration of the pectoralis major	CP, lost connection	No	CXR, chest-abdomen CT	Surgery	5 days	Inferior diaphragm, properitoneal space	No
Brignole et al. [15]	NR	NR	NR	BioMonitor 2, Biotronik	77.5 × 8.6 × 4.6 mm	NR	Excessive penetration angle	NR	NR	CXR	NR	NR	Posteroinferior recess	NR
Preminger et al. [16]	78 Male	HTN, hyperlipidaemia, OSA	29.8 kg/m^2^	Medtronic LINQ	44.8 × 7.2 × 4.0 mm	Left 4th intercostal space	Tip penetrated pectoralis major muscle	CP, SOB, lost connection	Migration after cardiac rehabilitation	CXR, CT	VATS	35 days	Anterior costophrenic angle	No

NR: not reported, CP: chest pain, CXR: chest X-ray, CT: computerised tomography, SOB: shortness of breath, HTN: hypertension, CKD: chronic kidney disease, AF: atrial fibrillation, OSA: obstructive sleep apnoea, VATS: video-assisted thoracoscopic surgery.

Several patterns and potential risk factors emerged from these cases, including patient-related, device and implantation-related, and post-implantation behaviours, monitoring and intervention-factors.

### 3.1 Patient-related factors

**Age:** Older age appears to be a common factor in ILR migration cases, with most patients in the reviewed cases aged over 60. Older patients may have increased risks due to age-related reduced tissue elasticity and slower healing rates [17]. Also, thin chest wall structure might lead to intrathoracic migration [18].
**Gender:** While the majority of patients in migration cases were female, broader studies report a slight male predominance, with 50–61 % of recipients being male [[2], [3], [4], [5], [6]]. This suggests that female gender may be a migration-related factor, potentially influenced by differences in soft tissue distribution that could influence device stability [19].
**Comorbidities:** Chronic conditions such as hypertension, dyslipidaemia, chronic kidney disease, chronic obstructive pulmonary disease, and malignancies might impact tissue integrity or healing [20], potentially increasing migration risk.
**Body Mass Index (BMI):** Although this was not consistently reported in the reviewed cases, it is hypothesised that patients with lower BMI could be more prone to migration due to less subcutaneous tissue. This aligns with findings from other implantable devices where minimal soft tissue provides less stability [21].

### 3.2 Device and implantation-related factors

**ILR Model and Size:** Larger devices (e.g., the BioMonitor series) were more frequently involved in migration cases. While larger devices might intuitively seem more stable, greater pressure on surrounding tissues, deeper implantation, or gravitational forces may increase the risk of migration.
**Implantation Technique and Site:** Devices implanted with greater angulation or deeper penetration into muscle tissue, particularly closer to the intercostal muscles, might carry a higher migration risk, as observed in some cases. If the penetration angle exceeds 45°, the pocket tool could accidentally pass through the intercostal space with a higher risk of migration [22]. Using a horizontal approach allowed for adequate sensing while reducing the risk of ILR migration [23].

### 3.3 Post-implantation behaviours, monitoring, and intervention

**Patient Activity and Device Manipulation:** Migration was observed in patients after reporting device palpation or physical activity, such as cardiac rehabilitation. Repeated muscle contractions likely contributed to device migration into the pleural space.
**Detection Time:** Detection times for migration in these cases ranged from as early as 5 days to up to 35 days post-implantation, with most migrations identified within the first month. This variation suggests that migration risk is often higher soon after implantation. Routine follow-up, ideally by the end of the first month, may help capture early migration cases before complications escalate.
**Signs of Migration:** Common signs of ILR migration included a loss of connection and chest pain. Loss of signal or connection was often the first indicator of migration. While chest pain was especially important for patients reporting continuous pain, which may indicate movement or friction of the device against surrounding structures. Hence, when an unusual or continuous pain occurs despite local anaesthesia a chest x-ray should be ordered.
**Migration Site:** Many devices shifted posteriorly, often into the pleural or costophrenic recess. This may be influenced by initial implantation angle, gravitational forces, or anatomical variations. These areas are naturally lower in position and more prone to gravitational forces [24].
**Intervention:** VATS was the most common intervention to remove the ILR. Although minimally invasive, VATS carries risks such as pneumothorax, infection, and prolonged recovery periods [25]. This highlights the importance of optimising ILR implantation techniques and post-operative surveillance.

This review is limited by the small sample size and reliance on case reports, which are subject to publication and reporting biases. Hence, the findings should be interpreted cautiously. Migration cases are likely underreported in the literature, particularly those with minor consequences that do not require surgical intervention. As such, the findings of this review may not fully capture the true incidence or spectrum of migration. Comparative data between patients with and without migration are needed to validate the identified factors.

## 4 Conclusion

Implantable loop recorder (ILR) migration represents a rare but important complication that can affect device efficacy, patient comfort, and healthcare resources. The requirement for VATS in severe cases shows the significant morbidity associated with this complication. While this review identifies potential factors associated with ILR migration, the findings should be interpreted cautiously due to the small sample size and potential reporting bias. Variability in reporting highlights the importance of standardised protocols for documenting ILR complications. Simultaneously, larger, prospective studies with standardised reporting are essential to validate these observations. Yet, based on this review, we suggest:

1. Conducting preoperative assessments considering BMI and comorbidities that may affect tissue stability.
2. Educating patients on avoiding behaviours (e.g., manipulation) that may lead to migration.
3. Establishing a follow-up protocol within the first month post-implantation to detect early signs of migration, especially in patients with continuous symptoms post-implantation.

## CRediT authorship contribution statement

**Allam Harfoush:** Writing – original draft, Project administration, Methodology, Investigation, Conceptualization.

## Ethical statement

This manuscript is a review of previously published case reports and does not involve any new data collection, human participants, or animal studies. All data reviewed and discussed within this paper are publicly available in the original publications, and appropriate citations have been provided. Consequently, no ethical approval or informed consent was required for this study.

## Declaration of Generative AI and AI-assisted technologies in the writing process

During the preparation of this work the author used (ChatGPT-4o) in order to improve the readability and language of the paper, and correct grammatical errors in certain sections. After using this tool, the author reviewed and edited the content as needed. The author confirms that no part of the thesis was generated by artificial intelligence.

## Funding

None received.

## Declaration of competing interest

The authors declare that they have no known competing financial interests or personal relationships that could have appeared to influence the work reported in this paper.
